# Diaphanous-Related Formin 2 and Profilin I Are Required for Gastrulation Cell Movements

**DOI:** 10.1371/journal.pone.0003439

**Published:** 2008-10-21

**Authors:** Shih-Lei Lai, Tun-Hao Chan, Meng-Ju Lin, Wei-Pang Huang, Show-Wan Lou, Shyh-Jye Lee

**Affiliations:** 1 Institute of Zoology, National Taiwan University, Taipei, Taiwan; 2 Department of Life Science, National Taiwan University, Taipei, Taiwan; 3 Institute of Fisheries Science, National Taiwan University, Taipei, Taiwan; Max Planck Institute of Molecular Cell Biology and Genetics, Germany

## Abstract

Intensive cellular movements occur during gastrulation. These cellular movements rely heavily on dynamic actin assembly. Rho with its associated proteins, including the Rho-activated formin, Diaphanous, are key regulators of actin assembly in cellular protrusion and migration. However, the function of Diaphanous in gastrulation cell movements remains unclear. To study the role of Diaphanous in gastrulation, we isolated a partial zebrafish diaphanous-related formin 2 (*zdia2*) clone with its N-terminal regulatory domains. The GTPase binding domain of zDia2 is highly conserved compared to its mammalian homologues. Using a yeast two-hybrid assay, we showed that zDia2 interacts with constitutively-active RhoA and Cdc42. The *zdia2* mRNAs were ubiquitously expressed during early embryonic development in zebrafish as determined by RT-PCR and whole-mount *in situ* hybridization analyses. Knockdown of *zdia2* by antisense morpholino oligonucleotides (MOs) blocked epiboly formation and convergent extension in a dose-dependent manner, whereas ectopic expression of a human *mdia* gene partially rescued these defects. Time-lapse recording further showed that bleb-like cellular processes of blastoderm marginal deep marginal cells and pseudopod-/filopod-like processes of prechordal plate cells and lateral cells were abolished in the *zdia2* morphants. Furthermore, zDia2 acts cell-autonomously since transplanted *zdia2*-knockdown cells exhibited low protrusive activity with aberrant migration in wild type host embryos. Lastly, co-injection of antisense MOs of *zdia2* and zebrafish *profilin I* (*zpfn 1*), but not zebrafish *profilin II*, resulted in a synergistic inhibition of gastrulation cell movements. These results suggest that zDia2 in conjunction with zPfn 1 are required for gastrulation cell movements in zebrafish.

## Introduction

In teleosts, an embryo rapidly cleaves from one cell into thousands of blastomeres. Following the cleavage period, the mass of blastomeres moves as a unit from the animal pole toward the vegetal pole to enclose the entire yolk in a process called epiboly. Gastrulation begins after the 50% epiboly stage when deep cells involute/ingress to form the mesendodermal layer, and convergence and extension follow to sculpt the body atlas [1], [2]. During gastrulation, tightly regulated cellular movements, which relies on the fast remodeling of the cytoskeleton, especially actin filaments, are required for proper development.

Dynamic remodeling of actin filaments has been heavily studied in cell division and cell migration. It involves several key cytoskeletal regulators. Among them, Rho-family GTPases take the center stage [3]–[5]. Rho GTPases, including Rho, Rac, and Cdc42, are small GTP-binding proteins with intrinsic GTPase activity. They cycle between active GTP-bound and inactive GDP-bound forms to regulate cytoskeleton-related cellular processes, including exocytosis, endocytosis, vesicle transport/secretion, cytokinesis, and cell migration [6]. Among Rho GTPases, Rho-dependent signaling has been most intensively studied. Rho functions through its downstream effectors, including Rho-associated kinase (ROCK), citron-kinase, and formin-homology (FH) proteins [4], [7], [8].

Formins are multi-domain proteins regulating dynamic remodeling of the cytoskeleton, and are best characterized by their conserved FH2 domain [9]–[12]. The FH2 domain has actin-nucleation activity and often forms dimers associated with F-actin barbed ends. The F-actin barbed end capping activity is due to the “leaky capper” property of FH2 [13]–[16], which causes rate reduction in both polymerization and depolymerization of F-actin. However, formins also prevent other tight capping proteins such as capZ homologues and gelsolin from tight-capping to barbed ends of F-actin. As a result, formins accelerate instead of attenuate actin nucleation [13]. In addition to FH2 domain, another two conserved FH domains are the proline-rich FH1 and FH3 domains. The FH1 domain binds to the SH3 domain of Profilin and cooperates with Profilin in actin nucleation [17]. The FH3 domain of yeast formin Fus1 has been shown to be involved in targeting Fus1 to the projection tip during conjugation [18]. The localization of a mammalian Diaphanous, mDia1, to the mitotic spindle was also found to be determined by the FH3 region in a study expressing GFP fusion constructs of various truncated mutants of mDia1 in Hela cells [19].

Members of a formin subfamily, the Diaphanous-related formins (Drfs), have a characteristic N-terminal Rho GTPase-binding domain (GBD), which binds intra-molecularly to its C-terminal Diaphanous autoregulatory domain (DAD) to impose autoinhibition activity through structurally blocking its own FH1-FH2 domains [20]–[23]. In cellular and *in vitro* binding studies, the binding of Rho to the Drf GBD domain exposes the FH2 domain and thus activates actin-nucleating activity [14], [17], [22], [24]. Although, the actin nucleating mechanisms and cellular functions of Drfs have been intensively studied, the roles of Drfs and other formins during embryonic development are still not well understood. FH proteins have been shown to play essential roles in cytokinesis in *Drosophila* [25] and *Caenorhabditis elegans* [26]. In addition, the mutation in human *diaphanous* causes defects in oogenesis and fertility [27], and the FH-domain-containing protein Daam1 has been shown to be required for the activation of Rho by Wnt/Frizzled signaling during gastrulation in *Xenopus lavis* [28]. However, the inhibition of cytokinesis induced by blocking the FH protein hinders its functional study at the later gastrulation stage. The dynamic cellular migration during gastrulation is also difficult to monitor in an opaque *X. lavis* embryo. As a result, the zebrafish is an excellent model to study the gastrulating cellular movement, since zebrafish embryos are transparent throughout early development. Furthermore, a popular morpholino oligonucleotide (MO) gene knockdown approach allows easy manipulation of gene activity in zebrafish [29]. Although MO is ineffective in blocking maternal protein activity, it is advantageous for allowing embryos to pass through the early cleavage period in the presence of MOs targeting cytokinesis-essential genes like Drfs. Therefore, the effects of Drfs on cell migration during gastrulation can be examined by applying the MO approach. We have identified a Diaphanous-related formin gene, *zdia2*, in zebrafish and found that it is ubiquitously expressed throughout early development. zDia2 was also shown to be essential along with the actin binding protein, profilin I, for epiboly formation and convergent extension, possibly via regulating gastrulating cell protrusive activities.

## Results

### Cloning, sequencing, and expression profile analysis of *zdia2*

**Figure 1:**
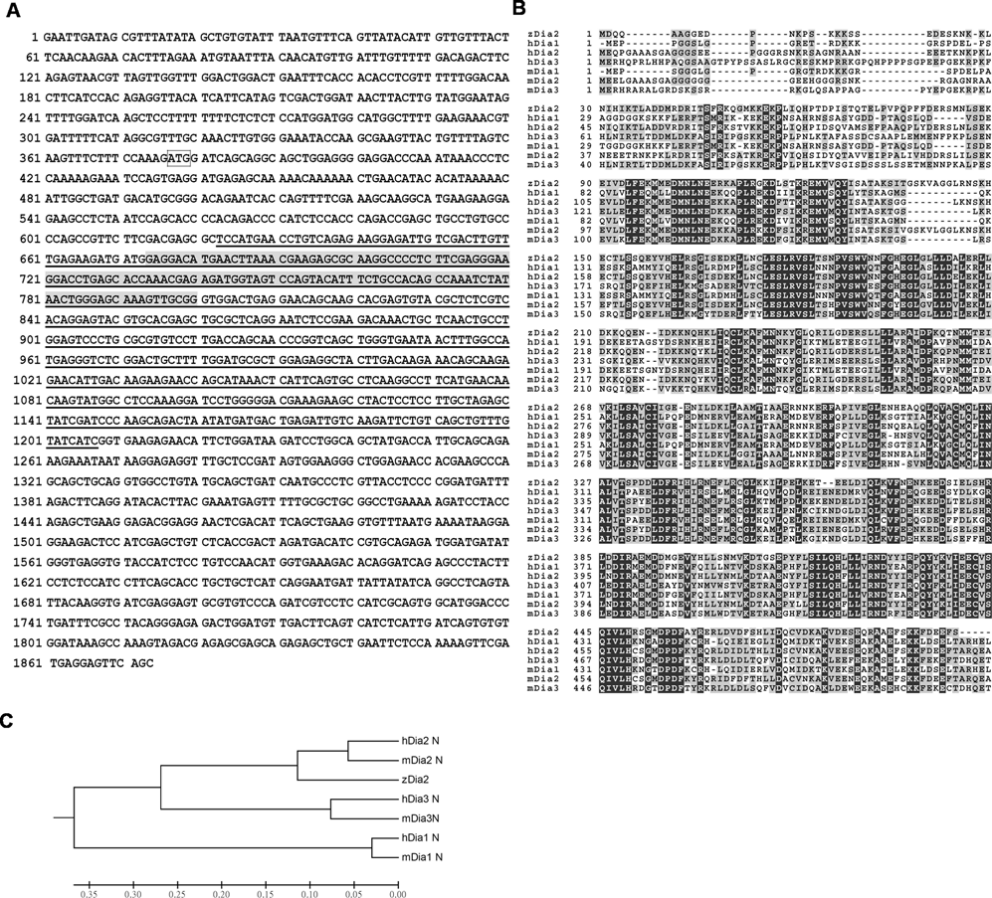
Sequence analysis of the zebrafish *diaphanous 2* gene (*zdia2*). (A) Nucleotide sequence of *zdia2*. A 5′-half zebrafish *dia2* sequence was cloned by 5′RACE. It contained 376-bp 5′UTR and 1497-bp coding regions (the start codon is boxed). A putative Rho-binding domain predicted by HMMPFAM is underlined. Exon 4, a putative deleting region by a *zdia2* splicing blocking MO, is shown with a gray background. (B) Amino acid sequence alignment of the cloned zDia2 with corresponding regions of human and mouse diaphanous-related formins 1, 2, and 3. Identical and conserved amino acids are shown with dark and gray backgrounds, respectively. (C) Phylogenic analysis of Dia members in chordates. The horizontal length is proportional to the estimated time from divergence of the gene from the related family member. h, human; m, mouse; z, zebrafish.

To investigate the roles of Diaphanous during embryonic development, we first cloned a *diaphanous* gene with the 5′ terminal partial sequence in zebrafish. We designed a pair of primers based on the zebrafish *diaphanous* gene sequence predicted by Ensemble and used it for PCR cloning. A 578-bp fragment (1295–1873 of Fig. 1A) with the predicted *diaphanous* sequence was obtained by PCR, and used to synthesize a riboprobe for whole-mount in situ hybridization (WISH) that will be described later. Using 5′-RACE, we further cloned a 1.86-kb PCR fragment, whose sequence revealed that it contained the 5′ terminal partial sequence with 376 bp of the 5′UTR region (Fig. 1A). Domain analysis of the translated amino acid sequence by HMMPFAM (http://workbench.sdsc.edu) revealed a putative GBD domain ranging from nucleotides 623 to 1207 and amino acids 83 to 277 of the partial sequence, respectively (Fig. 1A and B). Phylogenic analysis of the translated amino acid sequence further showed high sequence homology (48%∼80% in similarity) to the corresponding region of Dia2 in both the human and mouse (Fig. 1C). Therefore, we refer it as zebrafish *diaphanous 2* (*zdia2*, GeneBank accession no. **DQ979887**) here.

**Figure 2:**
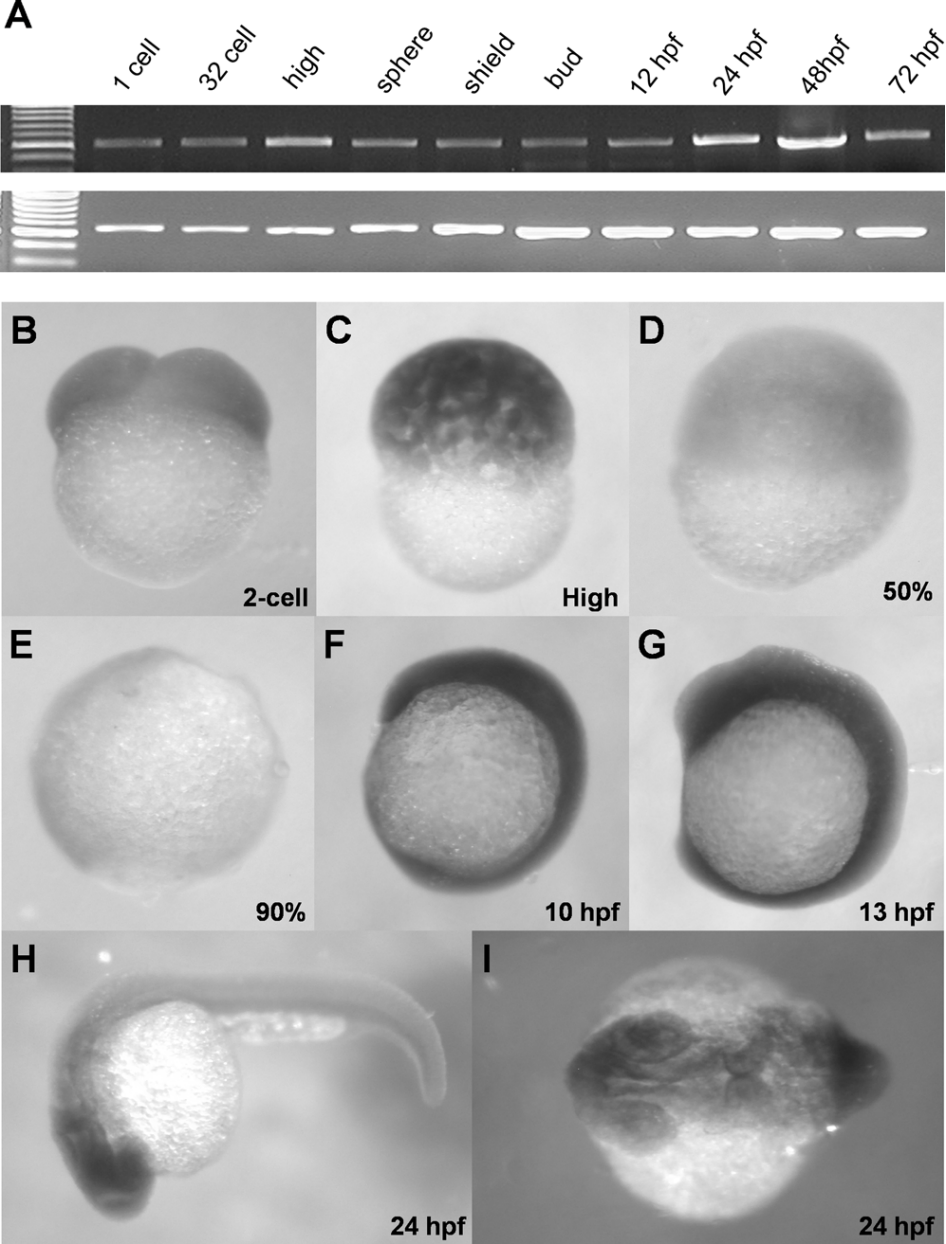
Ubiquitous expression of *zdia2* during early embryonic development. (A) Expression of a 578-bp fragment of *zdia2* at different embryonic stages was examined by RT-PCR, and the transcription of a 717-bp *β-actin* fragment was used as an internal control. (B-I) WISH of *zdia2* showed ubiquitous expression in the entire embryo during early stages, but the transcripts had enriched in the head region at 24 hpf. The riboprobe was synthesized from a 1487-bp fragment beginning at the start codon of *zdia2*.

To investigate the *zdia2* gene expression profile, we analyzed its mRNA expression during early development of zebrafish embryos by RT-PCR. In addition, the spatial and temporal localizations of *zdia2* mRNA were also examined by WISH. An antisense riboprobe against the 578-bp *zdia2* fragment with no GBD domain was used. This *zdia2* riboprobe does not hybridize the conserved GBD sequence that reduces the possibility of cross hybridization with other potential Diaphanous family genes in zebrafish. Both RT-PCR (Fig. 2A) and WISH (Fig. 2B–I) analyses showed that *zdia2* mRNA was present in all developmental stages examined. WISH results further revealed that *zdia2* mRNA was initially ubiquitously expressed in the entire embryo at least until 13 hpf, and appeared to be enriched in the head region at 24 hpf. The ubiquitous expression during early development suggests a universal role of zDia2 in regulating early embryogenesis.

### zDia2 specifically interacts with constitutively-active RhoA and Cdc42 but not Rac1

**Figure 3:**
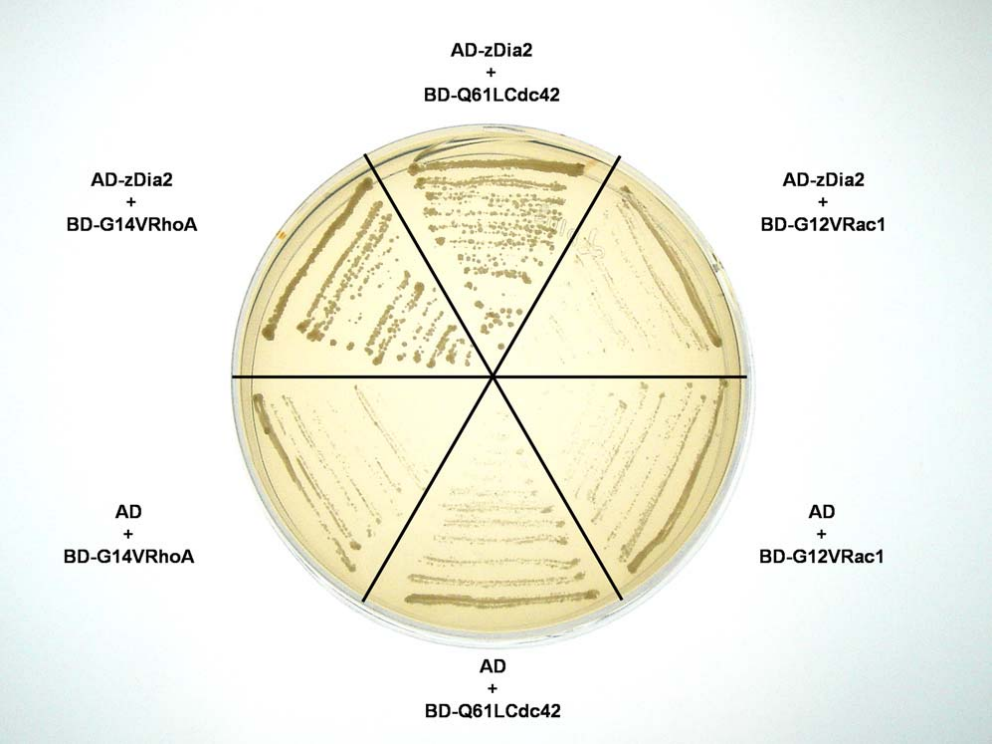
The zDia2 N-terminal half domain specifically interacted with constitutively active RhoA and Cdc42 in the yeast two-hybrid assay. Yeasts were transformed with designated constructs and tested for interaction by growth on selective medium lacking histidine with the addition of 4 mM 3AT. AD, GAL4 activation domain; BD, GAL4 DNA-binding domain.

To identify which active form of small GTPases may interact with the zDia2 GBD and possibly activate zDia2 as previously described in other systems [22], [30], we performed paired yeast two-hybrid assays to search for possible interactions between zDia2 GBD and Rho-family GTPases in zebrafish. Results showed that yeast co-transformed with *zdia2* and constitutively active G14VRhoA or Q61LCdc42 but not G12VRac1 grew on plates lacking histidine (Fig. 3). These results and previous studies [14], [17], [22], [24] on Diaphanous suggested that zDia2 interacts with constitutively-active RhoA and Cdc42 and might thus functions as their effectors during development.

### Knockdown of *zdia2* inhibits epiboly and convergent extension

**Figure 4:**
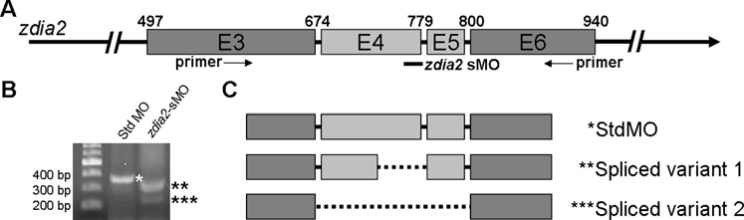
The *zdia2* splice-blocking morpholino oligonucleotide (sMO) caused splicing aberrance of *zdia2* exons 4 and 5. (A) Diagram of a partial pre-mRNA map of *zdia2*. The exons are shown in boxes, labeled with the corresponding exon number in E3 through E6, and the introns are represented by solid lines. A *zdia2* sMO target site is at the end of E4 to the beginning of following intron, which is indicated underneath as a black bar. The *zdia2* sMO would presumably interfere with the splicing of E4 shown in light grey. (B) The *zdia2* sMO induced splicing aberrance as evidenced in the RT-PCR analysis using a primer pair as indicated in (A) by arrows. Two spliced variants were found in *zdia2* sMO-treated embryos. The first variant (**) was 324 bp and the second variant (***) was 231 bp as compared to the 357-bp original transcript (*). (C) The spliced variants were sequenced (see Fig. S1) and shown as maps. The *zdia2* sMO-affected exons are shown in light grey and the deleted regions are depicted in dotted lines.

To study the roles of zDia2 during early embryogenesis, non-overlapping MOs blocking pre-mRNA splicing (sMO) or translation initiation (tMO) of *zdia2* were microinjected into zebrafish embryos to knock down *zdia2* gene expression. The *zdia2* sMO targets the 5′-splicing site of the *zdia2* fourth intron between exons 4 and 5 as indicated in Fig. 4A, which presumably would interfere with the splicing of exon 4. Elimination of exon 4 from mature *zdia* mRNA would delete amino acids 100∼135 (Fig. 1B), a highly conserved region for Rho binding [31], which is required for Rho-dependent activation. We used RT-PCR to confirm the effects of the *zdia2* sMO on *zdia2* pre-mRNA splicing. RNA isolated from control and *zdia2* sMO-injected embryos was analyzed by RT-PCR using primers targeting exons 3 and 6 as indicated by the arrows in Fig. 4A. PCR products were separated by agarose gel electrophoresis, gel-purified, and sequenced. A predicted 357-bp band (*) was amplified from the standard control MO (stdMO)-treated embryos. In contrast, the *zdia2* sMO-treated embryos showed two major spliced variants (Fig. 4B). Sequence analysis demonstrated that one fragment is a partial deletion of exon 4 (**), and the other fragment (***) lost both exons 4 and 5 (Fig. 4C and see sequences comparison in supplementary Fig. S1). No original 357-bp band was observed in the *zdia2* sMO-treated embryos, which confirms the effectiveness of *zdia2* sMO in blocking the splicing of *zdia2*.

**Figure 5:**
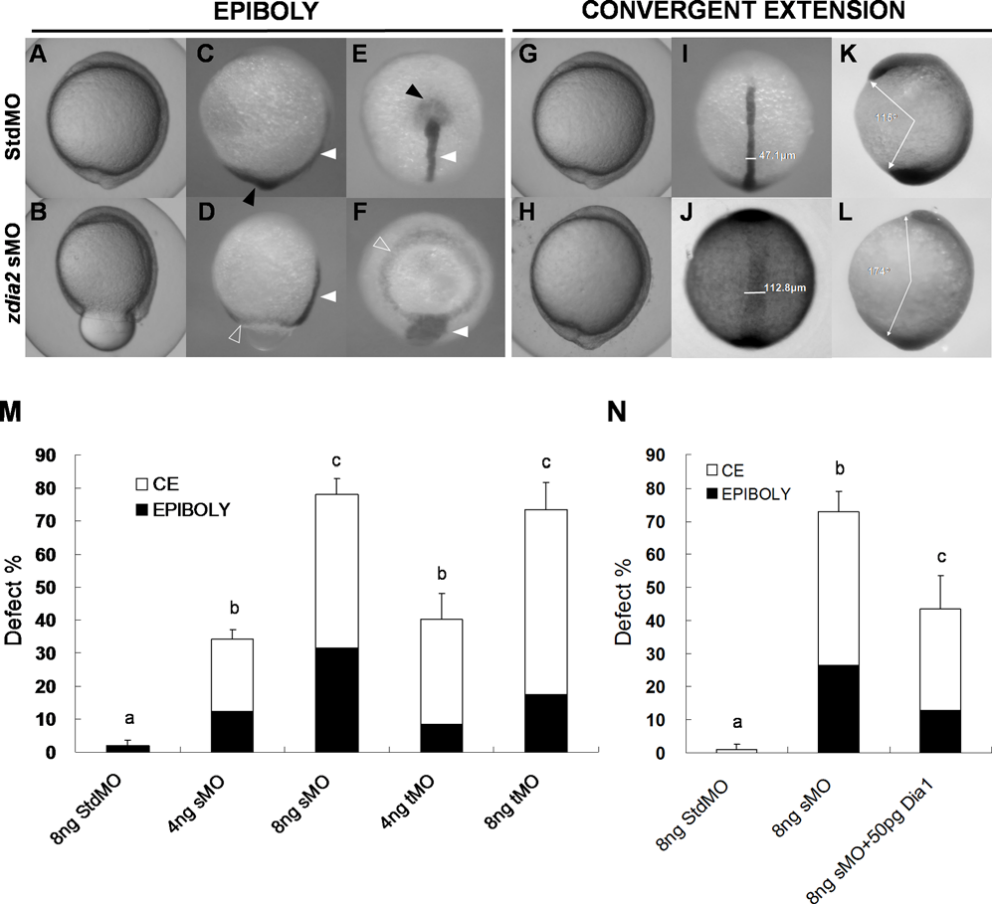
Knockdown of *zdia2* by MO interferes with gastrulation cell movements in a dose-dependent manner. Embryos injected with 8 ng of standard control (std) MO (the upper row) and *zdia2* sMO (the bottom row) were examined and photographed under a stereomicroscope at 10 hpf. Embryos were then fixed, stained by a *no tail* riboprobe (C, D, K and L, side view; E and F, vegetal view; I and J, dorsal view) or in combination with a *goosecoid* riboprobe and photographed (K and L, side view). Tail buds and notochords are respectively indicated by black and white arrowheads (C–F). The *zdia2* sMO caused incomplete epiboly formation with a ring structure near the vegetal pole as indicated by the *ntl* staining (D and F, open arrowheads). Widths of the notochords were measured and labeled in I and J. Lengths of the body axis were also estimated by the angle (labeled) between the prechordal plate and tail bud as described in the text (K and L). (M) Different MOs at 4 or 8 ng as designated were injected, and the embryos were examined at 10 hpf. Each treatment was repeated at least three times and analyzed as described, and only upper error bars of standard deviations are shown. (N) Embryos were injected with stdMO, *zdia2* sMO or *zdia2* sMO with 50 pg *mdia* mRNA as indicated. Epiboly and convergent extension defects were determined, analyzed, and presented as described in (M). Different letters on top of each column indicate a significant difference between treatments (P<0.05).

To examine the loss of function effects of *zdia2* on embryonic development, different amounts of MOs were injected into zebrafish embryos. The development of treated embryos was observed and photographed at designated times. No significant abnormalities were observed during cleavage stages in either the stdMO or *zdia2* sMO at 8 ng injected per embryo. Two kinds of phenotype correlating with severity of *zdia2* knockdown effects were observed, including epiboly arrest and convergent extension defect. The epibolic movement of the epithelial enveloping layer, along with the deep cells and dorsal forerunner cells of some *zdia2* MO-injected embryos slowed down and arrested at the 80%∼90% epiboly stage, and formed a huge yolk plug as shown in Fig. 5B. In contrast, the stdMO-injected embryos completed epiboly and formed normal head and tail buds at 10 hpf (bud stage, Fig. 5A), which had similar development to that of uninjected embryos (data not shown). To further examine the migration of blastoderm marginal deep cells, dorsal forerunner cells and subsequent notochord development, we applied WISH to those embryos using a *no tail* (*ntl*) riboprobe. The *ntl* riboprobe clearly marked the tail bud (Fig. 5C and E, black arrowheads) and the notochord (Fig. 5C–F, white arrowheads) in both stdMO- and sMO-injected embryos at 10 hpf (tail-bud stage). The stdMO-injected embryos showed thin and long notochord and a complete fused tail bud (Fig. 5C and E). In contrast, tail bud failed to form (originally formed by fusion of the deep marginal cells) and a blastoderm ring was shown in side (Fig. 5D, open arrowheads) and vegetal pole (Fig. 5F, open arrowhead) views, along with a wider notochord (Fig. 5F) in the 8 ng *zdia2* sMO-treated embryos. These results indicate that the marginal deep cells and dorsal forerunner cells of some *zdia2* sMO-injected embryos slowed down and finally stopped migrating at the 80–90% epiboly stage and were unable to fuse to form the tail bud at 10 hpf.

Convergent extension occurred during gastrulation to form a narrowed and elongated notochord and body axis as shown in stdMO-treated embryos at the tail bud stage (Fig. 5G and I). For those *zdia2* sMO-treated embryos, which completed epiboly, their convergent extension movements were obviously interfered that resulted in a broader notochord (Fig. 5J vs. Fig. 5I), and a less-extended body axis compared to the stdMO-injected ones (Fig. 5L vs. Fig. 5K) at 10 hpf. To more precisely measure the effect of *zdia2* sMO on the extension of the body axis, a *goosecoid* (*gsc*) riboprobe was used to mark the prechordal plate (the presumptive head bud) in conjunction with labeling of the notochord and tail bud by the *ntl* riboprobe. The extension of the body axis was estimated by measuring the angle formed by drawing white arrows connecting the front and back edges of the head and tail bud, respectively, to the center of the yolk as shown in Fig. 5K and L. A larger inward angle indicates shorter body axis. The *zdia2* sMO resulted in a notably reduced extension of the body axis with a larger inward angle in the treated embryos.

The *ntl* and *gsc* riboprobe staining allowed us to measure the width of the notochord and the length of the body axis as estimated by the degree of body surrounding the yolk at the tail-bud stage. These parameters provide fine criteria for estimating convergent extension cell movements during gastrulation. Therefore, we used them to measure the notochord width and the body axis length in embryos labeled with *ntl* and *gsc* riboprobes in each group for quantitative analyses. Compared to the stdMO-injected embryos, broadened notochords at the tail-bud stage were observed in *zdia2* sMO morphants. The notochord width of these *zdia2* sMO morphants (112.8±1.4 µm, *n* = 72) was significantly larger than that of stdMO-treated embryos (47.1±3.6 µm, *n* = 74). In addition, these *zdia2* sMO morphants had a shorter body axis (173.7°±3.1°, *n* = 76) as compared to stdMO-treated embryos (115.1°±5.2°, *n* = 99). Similar results on the inhibition of epiboly formation and convergent extension were also obtained by injecting a translation-blocking MO (tMO) at the same dosage (data not shown). These results demonstrated the effects of *zdia2* sMO on the cellular movements of *zdia2* morphants during gastrulation.

To measure the dose-dependent effects, we injected different amounts of *zdia2* MOs or stdMO into 1-cell embryos and monitored the effects on epiboly formation and convergent extension. Quantitative analysis in three independent experiments showed that the defects caused by *zdia2* sMO and a tMO were dose-dependent (Fig. 5M). StdMO at 8 ng caused very mild effects on gastrulation, which resulted in 1.9% defect rate in epiboly and no convergent-extension defect were observed (*n* = 106). The defects in epiboly and convergent extension were significantly (p<0.05) and dose-dependently increased in the 4 ng (n = 96) to 8 ng (n = 113) *zdia2* sMO-injected embryos. Similar dose-dependent inhibition were observed in the embryos injected with 4 ng (*n* = 106) and 8 ng *zdia2* tMO (n = 99) (Fig. 5M). To further examine whether the observed the convergent extension defects were due to the effects of developmental retardation, we fixed embryos at 6∼8-somite stages, labeled them with a myogenic factor, *myoD* riboprobe by WISH, and measured somites widths of those embryos. Embryos injected with *zdia2* sMO had significantly wider somites (172.4±20.6 µm) compared to stdMO-injected embryos (128.6±8.3 µm) and shorter body axis (96.4°±10.3° extension angle) compared to stdMO-injected embryos (64.7°±5.5° extension angle) at this stage (Supplementary Fig. S2). It appeared that the convergent extension defects observed are not because of retarded development.

To further confirm the specificity of *zdia2* knockdown-resulted phenotypes, we performed functional rescue experiments by co-injecting *mdia* mRNA, which has been shown to rescue convergent extension defects in RhoA morphants in zebrafish [32]. Embryos co-injected with 50 pg *mdia* mRNA in addition to 8 ng *zdia2* sMO showed significantly reduced defect rates in both epiboly (12.8%) and convergent extension (30.8%) compared to those of 8 ng *zdia2* sMO-injected embryos (26.4% and 46.7%, Fig. 5N). The functional rescue experiment clearly indicated the *zdia2* morphant phenotypes were specifically due to the loss of Diaphanous activity.

### zDia2 regulates gastrulation cell movements via regulating cell protrusive activity

**Figure 6:**
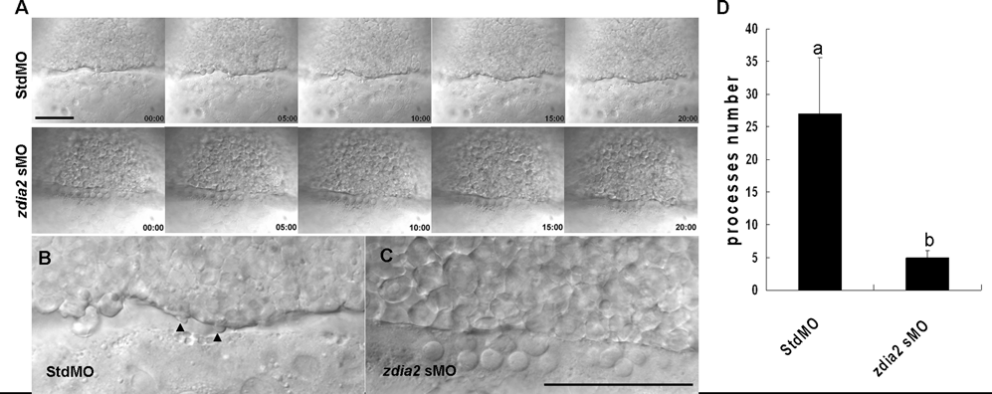
Knockdown of *zdia2* inhibits epiboly cell migration by inhibiting cell protrusion formation. Embryos injected with the standard control MO (stdMO) or the *zdia2* splice-blocking MO (sMO) were dechorionated, immobilized, monitored, and recorded on 20-minutes continuous time-lapse movies (See supplement movie S1 and S2 for the stdMO and sMO-injected embryo, respectively). Photographs from each recording at 5-minutes intervals are shown in (A) with the recording time given in the lower right corner. All photographs are positioned with animal pole up. In the magnified pictures, marginal deep cells of the stdMO-injected embryo (B) exhibited clear protrusive activity with two protruded cell processes indicated by arrowheads, but no such protrusions were evident in the *zdia2* sMO-treated embryo (C). Bars indicate 100 µm. (D) Cell protrusions formation at the leading edge of marginal deep cells in both stdMO- and *zdia2* sMO-injected embryos were quantified separately by counting protrusions formed within the first 50 frames form 3 different movies, respectively. Results were then analyzed and only upper error bars of standard deviations are shown. Different letters on top of each column indicate a significant difference between treatments (P<0.05).

To investigate the cellular mechanisms that underlie the cell movement defects in *zdia2* morphants, we used time-lapse CCD camera recording to observe cell behavior and protrusive activities in both stdMO and *zdia2* morphants. A region of the marginal deep cells from the side view was monitored during germ ring-70% epiboly stage, when active cell protrusive activity were observed in these cells of control embryos, and a 20-min movie of 10-s intervals was recorded (Fig. 6A and supplementary movie S1 and S2). First, marginal deep cells were found to migrate significantly faster (26.1±3.1 µm, n = 4) in the control embryos (see supplemental movie S1) compared to those of the *zdia2* sMO morphant in 20 minutes (14.8±4.1 µm, n = 6; see supplemental movie S2). Second, we found that these cells in control embryos formed bleb-like (bleb, hereafter) cell processes and the formation of these processes was obviously decreased in the *zdia2* morphants (Fig. 6C), compared to those in the stdMO-injected embryos (Fig. 6B). We quantified the blebbing cell processes formed within 50 frames in 500 sec from 3 movies each of the stdMO-injected embryos and *zdia2* MO-injected embryos. The results showed that marginal deep cells of stdMO-injected embryos formed significantly more blebbing cell processes, with an average of 27±8.5 processes/embryo, than those injected with *zdia2* sMO with only 5±1 processes/embryo. To examine this more precisely, we labeled blastomere membrane by co-injecting mRNA of membrane-associated protein GFP-GAP43 [33] with stdMO or *zdia2* MO and observed those injected embryos under confocal microscope. It appeared that the position and shape of those blebbing cell processes revealed by membrane-associated GFP-GAP43 proteins were coincided to their DIC images (supplementary Fig. S3, and movie S3 and S4). As previously found in the DIC time-lapse recordings (supplementary movie S1 and S2), the marginal deep cells migrated slower and formed notably less blebbing cell processes in the *zdia2* morphants compared to the control ones. These results provide a possible mechanism of how zDia2 functions in gastrulation cell movements.

**Figure 7:**
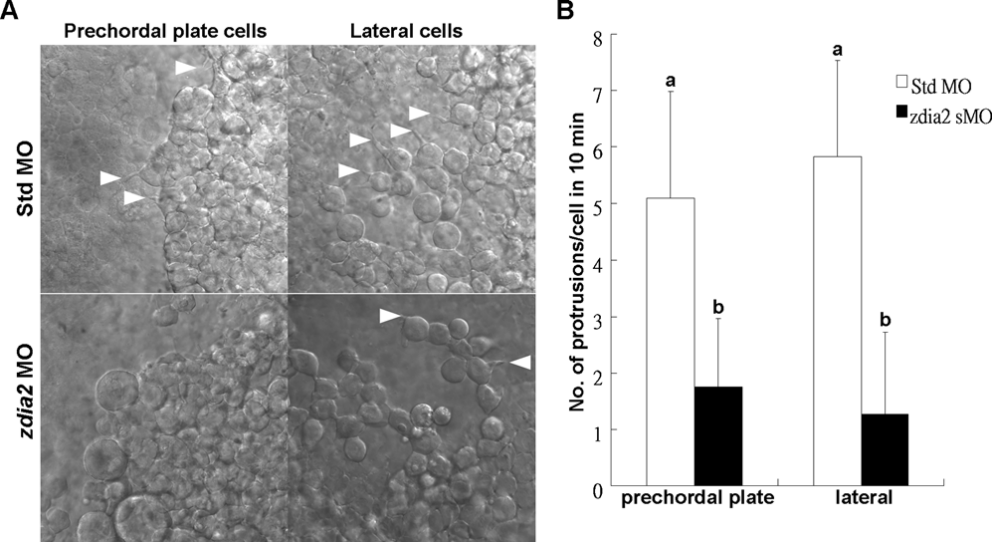
Knockdown of *zdia2* inhibits cell protrusion formation of prechordal plate (primordial) cells and lateral (mesendodermal) cells. (A) Embryos injected with the standard control MO (stdMO) or the *zdia2* splice-blocking MO (sMO) were dechorionated, immobilized, monitored, and recorded on 10-minutes continuous time-lapse movies. (B) Protrusions formation of cells on focus in both stdMO- and *zdia2* sMO-injected embryos were quantified separately by counting protrusions formed within the first 10 minutes form at least 3 different movies, respectively. Results were then analyzed and only upper error bars of standard deviations are shown. Different letters on top of each column indicate a significant difference between treatments (P<0.05).

In addition to blebbing, we also examined pseudopod-/filopod-like processes formed in prechordal plate (progenitor) cells and lateral (mesendodermal) cells, which involute from the dorsal and lateral side of the shield, respectively, right after germ-ring stage. Both types of cells migrated anteriorly toward the animal pole and dorsally toward the future axis in a process called dorsal convergence. We found that both prechordal plat cells and lateral cells formed significantly less protrusions, including both pseudopod-/filopod-like processes in *zdia2* morphants compared to that in control embryos (Fig. 7A). Prechordal plate cells and lateral cells formed 5.09±1.9 and 5.83±1. 7 protrusions, respectively, in the control embryos, but only 1.75±1.22 and 1.27±1.46 protrusions, respectively, in *zdia2* morphants within 10 min (Fig. 7B). Taken together, *zdia2* is required for the formation of cellular process in all types of cell examined throughout gastrulation and thus affects cell movements such as epiboly, dorsal convergence and convergent extension.

### Knockdown of *zdia2* blocks actin assembly in marginal deep cells and YSL

**Figure 8:**
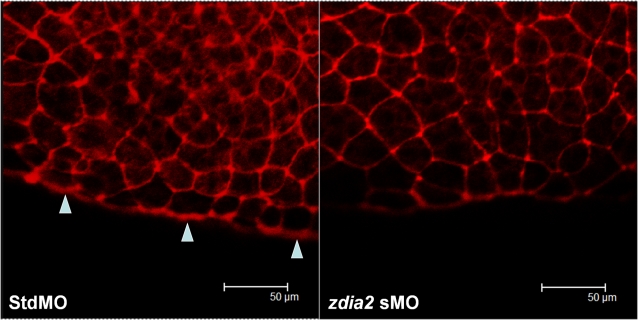
Knockdown of *zdia2* inhibit actin condensation at the front edge of blastoderm. Embryos injected with 8 ng stdMO and *zdia2* sMO were fixed at the germ-ring stage and photographed under confocal microscope after rhodamine phalloidin staining. Filamentous actins (arrowheads) were enriched at the front edge of the epiboly marginal deep cells of the stdMO-injected embryo (A), but not in the *zdia2* sMO treated one (B).

Cell protrusive activity is mainly controlled by actin filament dynamics and Diaphanous-related formins have been shown to regulate actin polymerization. Thus, we attempted to compare F-actin structure of *zdia2* morphants to stdMO embryos in gastrulating zebrafish embryos, and rhodamine phalloidin was used to stain actin filaments. In two independent experiments, we found that after 50% epiboly stage cortical actin condensed and assembled in the marginal deep cells that was similar as described by Cheng et al. [34] (Fig. 8A, arrowheads, n>10). In contrast, this structure was not observed in *zdia2* morphants (Fig. 8B, n>10). In addition, another ring-like actin cable in between enveloping layer (EVL) and yolk syncytial layer (YSL) has also been shown to be crucial for EVL epiboly during later epibolic stages. We compared phalloidin staining pattern between epiboly-defected *zdia2* morphants and control embryos with similar degree of epiboly (about 90% epiboly) stages and found that these *zdia2* morphants were completely lacking this YSL actin structure (Supplementary Fig. S4). EVL epiboly was disrupted as well as deep cells epiboly, probably as a result of losing this crucial actin structure. These results suggest that zDia2 plays a role in regulating epiboly cell movements at least partly through regulating actin dynamics to form specific F-actin structures, which might affect protrusive activity, especially the membrane blebbing we previously observed, and other functions such as conducting actomyosin contractility at the marginal EVL at later epibolic stages [35].

### *zdia2* function is required cell-autonomously for gastrulation cell migration

**Figure 9:**
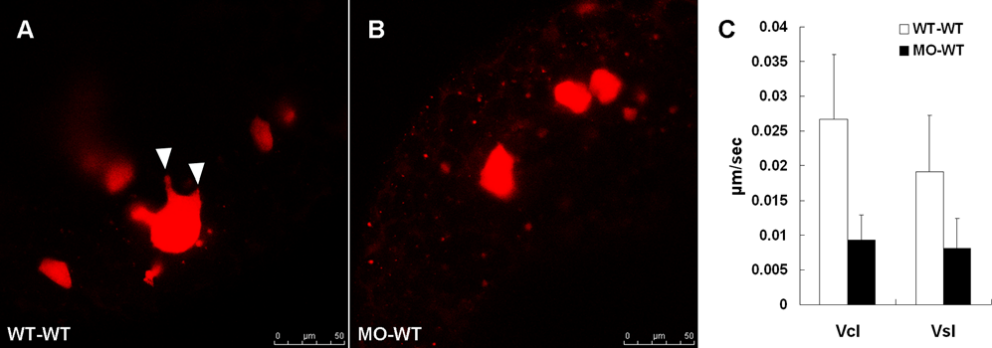
*zdia2* function is required cell-autonomously for the cell protrusions and migration. Rhodamine-labeled cells were transplanted from embryos injected with 8 ng StdMO (A) and 8 ng *zdia2* sMO (B) with Q-rhodamine, respectively, to wild type embryos. (C) Curvillinear velocity (Vcl) and strait line velocity (Vsl) were calculated and quantified (p<0.05).

To evaluate whether the *zdia2* function is cell-autonomous, we transplanted blastoderm cells from embryos, which were co-injected with stdMO or *zdia2* sMO with Q-rhodamine, which was used as a cell tracking marker, into wild type embryos and then monitored the morphology and movements of these cells under epifluorescent microscope. Those transplanted stdMO-treated cells formed protrusions more frequently compared to that of cells from *zdia2*-morphants. To obtain a clear photograph of those protrusive cells, we performed confocal microscopy and representative snapshots were taken to show that transplanted control cells indeed formed protrusions (Fig. 9A), but not in the *zdia2*-knockdown cells (Fig. 9B). Furthermore, we found that those stdMO-treated cells traveled with significantly higher speed with higher linearity and longer travel distance than those cells of *zdia2*-morphants when transplanted into wild type host embryos (Fig. 9C). The transplanted *zdia2*-morphant cells had lower curvilinear velocity (Vcl, curvilinear distance/time) of only 0.0093 µm/sec compared to the Vcl of transplanted StdMO-treated cells of 0.0267 µm/sec. In addition, the transplanted *zdia2*-morphant cells also migrated less linearly as shown by a lower straight line velocity (Vsl, straight line distance/time) of only 0.0081 µm/sec compared to the Vsl of transplanted stdMO-treated cells of 0.0192 µm/sec. Lastly, the transplanted *zdia2*-morphant cells actually migrated for a shorter distance in our recording (data not shown). These results indicate that *zdia2* is required for gastrulation cell migration in a cell-autonomous fashion.

### *zdia2* regulates gastrulation cell migration in coordination with profilin I but not profilin II

**Figure 10:**
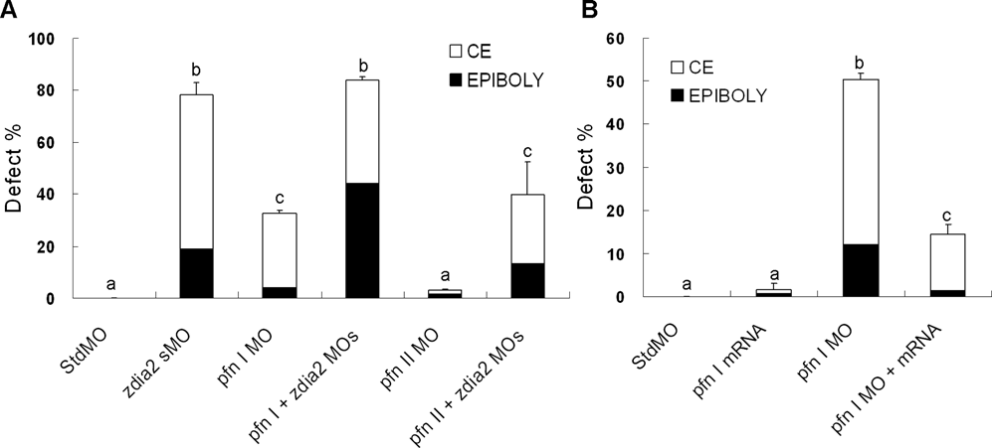
zDia2 coordinates with profilin I in the regulation of gastrulation cell movements. (A) The stdMO, *zdia2* sMO, *pfn I* tMO, or *pfn II* tMO were injected at 8 ng per embryo as indicated, while 4 ng *zdia2* sMO plus 4 ng *pfn I* MO (MOs(*pfn I*+*zdia2*)) or *pfn II* MO (MOs(*pfn II*+*zdia2*)) were co-injected as shown. Each treatment was repeated at least three times and the results were then analyzed by student t test. The defects are categorized as in Fig. 6, and only upper error bars are shown. (B) Defect rates of embryos injected with 8 ng stdMO, *pfn I* MO, *pfn I* mRNA at 100 pg and 8 ng *pfn I* MO plus 100 pg *pfn I* mRNA were quantified as designated, and the embryos were examined by the same criterion as mentioned previously at 10 hpf. The results were analyzed by student t test., and only upper error bars are shown. Different letters on top of each column indicate a significant difference between treatments (P<0.05).

To further investigate whether downstream targets of zDia2 are involved in actin dynamics during the process, we explored the possible involvement of profilins, a group of actin-binding proteins which are well-known in cooperating with mDia to promote actin polymerization [17]. We identified two profilin genes in zebrafish with sequence homology to mammalian *profilin I* and *II*, respectively. To analyze the roles of zebrafish *profilin I* (*zpfnI*) and *II* (*zpfnII*) in zebrafish gastrulation, we injected tMOs against *zpfnI* and *zpfnII*, respectively. Knockdown of *zpfnI* by 8 ng of *zpfnI* tMO (Fig. 10) caused 28.4% epiboly defect rate and 28.4% convergent extension defect rate (*n* = 122), which were milder to the percent defects induced by the same amount of the *zdia2* sMO. On the other hand, 8 ng of the *zpfnII* tMO had almost no effect on gastrulation with 1.6% (*n* = 121) and 1.7% (*n* = 121) defect rates for epiboly and convergent extension, respectively. Phenotypes caused by *zpfnI* tMO were indistinguishable from those of *zdia2* morphants (data not shown). To explore the possible interactive function of zDia2 and profilins, 4 ng *zdia2* sMO was co-injected with 4 ng of either the *zpfnI* or *zpfnII* tMO, respectively. Synergistic inhibition on epiboly and convergent extension was observed in the *zdia2* and *zpfnI* MO co-injected group, which were 44% (*n* = 128) and 39.9% (*n* = 128) defect rates in epiboly and convergent extension, respectively. These inhibitions were even more severe than those embryos injected with 8 ng of *zdia2* MO or the *zpfnI* MO alone. The percentage of epiboly-defective embryos increased more than two folds compared to that of 8 ng *zdia2* sMO- or 8 ng *zpfnI* MO-injected morphants (44% compared to 19% and 4.1%, respectively). Moreover, double-knockdown morphants showed even broader notochords and shorter body axis than *zdia2* morphants morphologically (Supplementary Fig. S5). In contrast, no such effect was observed by co-injecting *zdia2* and *zpfnII* MOs, which caused only 13.3% (*n* = 128) and 26.4% (*n* = 128) in epiboly and convergent extension defects, respectively, that resembled the defect rates caused by 4 ng *zdia2* sMO-injection along. Furthermore, defects caused by *zpfnI* MO could be rescued by co-expressing *zpfnI* mRNA. Both stdMO and *zpfnI* mRNA injection caused very low defect rates in epiboly and convergent extension. Embryos injected with *zpfnI* MO had 12.1% and 38.3% defect rates in epiboly and convergent extension, and these defects could be rescued by co-injecting 100 pg *zpfnI* mRNA. The co-injected embryos showed only 1.5% and 12.9% in epiboly and convergent extension, respectively (Fig. 10B). These results showed that zDia2 and zPfnI synergistically mediates the gastrulation cell movement in zebrafish.

## Discussion

### zDia2 is required for gastrulation cell movements in zebrafish

Rho is a key molecular switch during early embryonic development in both vertebrate and invertebrate embryos [36]–[39]. We previously showed that Rho mediates cytokinesis and epiboly cell migration in zebrafish via ROCK, probably through regulating the contractile activity of actin filaments [40]. Herein, we further examined the roles of another Rho downstream effector, Diaphanous-related formin, in zebrafish embryonic development. We (1) identified a zebrafish Diaphanous zDia2 and showed its interactions with constitutively-active RhoA and Cdc42, which implicated that zDia2 is a *bona fide* Rho GTPases effector, (2) showed that knockdown of zDia2 inhibits the progressions of epiboly, convergence and extension, and these defects could be functionally rescued by ectopic expression of human mDia, which further strengthen the conservation of Dia and its role in mediating gastrulation cell movements, (3) revealed that zDia MOs-induced gastrulation defects may be mediated via perturbation of actin filament assembly and cell protrusive activity at the leading edge of the blastoderm, lateral an prechordal plate cells, (4) observed that the function of *zdia2* is required cell-autonomously for gastrulation cell migration, and (5) demonstrated that the z*pfinI*, but not the z*pfinII*, MO exerts synergistic effects with the *zdia2* MO on the inhibition of gastrulation in zebrafish. These results clearly suggest that zDia2 plays an essential role in mediating Rho-dependent actin remodeling during zebrafish gastrulation.

### zDia2 regulates protrusive activity of marginal deep cells, prechordal plate progenitor cells and lateral mesendodermal cells and its role in gastrulation

According to our observations, active protrusive activity is required for various types of cell movement during gastrulation. To begin with, membrane blebbing occurred at the front row of marginal deep cells and oriented to migrating direction in zebrafish around germ-ring stage. However, this blebbing activity was significantly decreased in the *zdia2* morphants (Fig. 6, Fig. S3 and supplementary movie S1, S2, S3 and S4). It appeared that knockdown of *zdia2* only affected the formation of these blebs, but not the stability or orientation (see supplementary movie S1–4). Membrane blebbing is the first sign of cell motility when non-motile blastomeres transform into motile blastula cells. Blebbing has been suggested to be one type of crucial cell protrusions formed after mid-blastula transition during embryogenesis in vertebrates. Along with filolamellipodia, these cell protrusions serve as organs of locomotion [41], [42]. In the deep cells of killifish *Fundulus heteroclitus*, blebbing locomotion has been shown to be the driving force for directional cell migration during normal embryogenesis and in the wound healing process on yolk sac [43]. The blebbing locomotion is non-contact inhibiting and presumably could provide free space between neighboring deep cells for ingression and intercalation for cell migration during epiboly [44]. Indeed, instead of involuting as a flowing cohesive sheet, ingression of superficial deep cells has been shown to be the process occurring at the marginal blastoderm during germ ring stage in killifish that is preceded by blebbing locomotion [45]. Membrane blebbing has also been suggested to be important in cell migration and cancer cell invasion (See a review by Fackler and Grosse, 2008) [46]. Diaphanous has been shown to be crucial for plasma membrane remodeling, to be more specific, blebbing formation/retraction. Blebbing cell motility of human MDA-MB-435 cancer cells in 3D matrice relies on the action of the RhoA interaction partner and regulator Dia1 [47]. FHOD1, another functional similar formin, contributes to plasma membrane blebbing and enhances invasiveness [48]. In zebrafish, polarized blebbing is also important for primordial germ cells migration, which SDF-1 and CXCR4 activates myosin 2 and unknown actin nucleator by increasing calcium level at the site of migration direction [49]. In consistent with these observations, our results demonstrated that this blebbing locomotion serves to drive the marginal deep cell migration and is regulated by *zdia2*, since knockdown of *zdia2* led to the reduction of membrane blebbing and marginal deep cell velocity. These novel findings contribute to our understanding in the molecular regulation governing actin-mediated blebbing dynamics *in vivo*. In addition, pseudopods-/filopods-like protrusions in prechordal plate progenitor cells and lateral mesendodermal cells were also examined in this study. Previously, the formation and orientation of cellular processes of lateral cells [50]–[52] and prechordal plate cells [53], [54] have been addressed intensively. Our results showed that knockdown of zdia2 significantly decrease the formation of these cellular protrusions (Fig. 7) that results in decreased velocity and linearity of cell migration (Fig. 9). In this logic, the convergent extension defects observed in *zdia2* morphants would be possibly due to the disturbed anterior migration of mesendodermal cells and intercalation of cells align medio-lateral axis as revealed by a shorter prechordal plate-to-tail bud axis, a wider notochord and lateral expanded somites from WISH patterns of *gsc*, *ntl*, and *myoD* (Fig. 5 and Fig. S3). Collectively, these observations support a role of various types of cellular protrusions in gastrulation cell movements, but its mechanistic regulation requires further investigation.

### Actin-dynamics in epibolic cell movement

Previously, Cheng and colleagues uncovered at least three types of actin-based structures formed after the 50% epiboly stage [34]. Two of them are ring-like structures which form at the interface between EVL and YSL and the margin of deep cells. These actin structures are essential for the completion of epiboly, since the progression of epiboly can be interfered by disrupting actin rings using cytochalasin B. The actin condensation at the front margin of deep cells we observed in this study is more like the so-called “mesh-like network” of cortical microfilaments described in Cheng's study. This mesh-like network has been described in the deep cells of killifish gastrula [55] and has been reported to play a major role in most crawling cells [56]. In addition, the thicker ring-like actin band recruited at the interface of EVL and YSL starting after 50% epiboly was also examined in this study. By knocking down *zdia2* using MOs, we observed both a notable reduction in this F-actin condensation at the leading edge of the marginal deep cells, and abolished recruitment of actin band at YSL. This might lead to two possible outcomes, including abolishment of cell blebbing and prevention of F-actin mesh-like network constriction for epibolic movement. Subsequently, its cooperation with myosin via a so-called “purse-string mechanism”, which has been described in similar gastrulation processes such as dorsal closure in *Drosophila* [57], ventral closure in *C. elegans* [58], and possibly in epiboly of deep cells in zebrafish [59], would also be suppressed. Theses results confirm the presence of these F-actin structures around the margin of migrating blastoderm and their importance in epibolic movement. We also provide further evidence to show that zDia2 is necessary for the integrity of these F-actin structures. In addition, the necessity of Diaphanous gains further support in a recent report showing that Diaphanous is essential for contractile actomyosin assembly and stabilization during gastrulation in *Drosophila* [60]. Prior study revealed some underlie mechanism for the formation of this ring-like actin condensation at YSL in zebrafish [35]. Ste20-like kinase Msn1 seems to recruit actin and myosin 2 within the yolk cytoplasm and knockdown of Msn1 not only disrupts the formation of this actin condensation, but also affects the coordination of cell shape changes of marginal EVL cells and finally embryo epiboly. Phenotypes of *zdia2* morphants share similarities with *msn1* morphants, such as the disruption of YSL actin ring, and the slowdown/stop of epiboly of both deep cells and EVL at later epibolic stages (show defects only after 50% epiboly, in supporting of the requirement of the actin structure at later epiboly and other mechanisms underlie the early epiboly process, such as radial intercalation of deep cells). The detail molecular mechanisms controlling actin function in the YSL based on these findings still need further study.

### Upstream regulators of zDia2 in gastrulation cell movements

Several signaling pathways have been described as being involved in gastrulation cell movements, including non-canonical Wnt [28], hyaluronic acid-synthesizing enzyme 2 (Has2) [50], trimeric G protein subunits Gα12 and Gα13 [61], and Fyn/Yes signaling pathways [62]. Interestingly, they all somehow converge to the Rho-family GTPases pathways which suggest a fundamental role of Rho-GTPase signaling during gastrulation. Among them, non-canonical Wnt signaling seems to be most related to the gastrulation phenotypes we observed in this study. Zebrafish Wnt11 expresses at the epiblast cells of the germ ring, which correspond to the position of marginal deep cells we observed in this study. Wnts are a family of secreted molecules, which signal and stimulate surrounding cells. Zebrafish Wnt11 mutant *silberblick* shows epiboly delay during early gastrulation, especially the anterior migration of prechordal plate cells and the posterior migration of axial chordal plate cells, as well as the later convergent extension defects, which are very similar to what we have observed in the *zdia2* morphants [53], [63]. Moreover, Wnt has been shown to activate both RhoA and Rac through Dishevelled and Dishevelled-activated activator of morphogenesis (Daam1) during vertebrate gastrulation [28], [64]. Zhu and colleagues further demonstrated that overexpression of *mdia* and *rhoA* could rescue RhoA, and Wnt11 morphants, respectively, from convergence and extension defects in zebrafish [32]. We thus hypothesized that Diaphanous acts downstream of Wnt and RhoA during gastrulation in zebrafish. Our findings support the hypothesis by showing that zDia2 regulates actin nucleation and cell protrusive activity and expression of this *mdia* functionally rescued gastrulation defects caused by *zdia2* MOs in our hands. We further revealed that zDia may exert its function via the actin binding protein Profilin I. Taken together, the RhoA-mDia-Profilin signaling may be as suggested play an important role in controlling gastrulation under non-canonical Wnt signaling in zebrafish [64].

It is not surprising that Diaphanous interacts and being activated by more than one small GTPases. mDia2 interacts with both RhoA and Cdc42 and these interactions are crucial for the activation of mDia2 and the subsequent alteration of cell morphology and the formation of cell protrusions [65]. The preferential interaction of zDia2 with constitutively active RhoA and Cdc42 (Fig. 3) and its high sequence homology with mDia suggest there may be structural conservation between mammalian and teleost Dia2. Whether zDia2 is regulated by RhoA and/or Cdc42 during gastrulation still needs to be elucidated.

### zDia2 controls gastrulation cell movements in coordination with profilin I but not profilin II

Profilins are actin-binding proteins, which are highly conserved throughout the animal and plant kingdoms. Their conservation does not lie in sequence homologies but rather in protein folding between isoforms and species [66]. Profilins seem to have been functionally conserved between species since complementary experiments were successful in *Dictyostelium discoideum* using both plant and bovine profilins [67], [68]. The most essential function of profilin is the nucleotide-exchange activity that accelerates the ADP-ATP exchange of G-actin and replenishes the polymerization-ready ATP-actin pool in cells [69], [70]. However, different profilin isoforms seem to play different roles in tissue-specific manners. For example, *profilin I* is expressed throughout all embryonic stages and almost all adult cell types and tissues except skeletal muscle, while *profilin II* is exclusively expressed in the nervous system of mammals [71], [72]. Zebrafish contains both *profilin I* and *II*, which are expressed in similar ways to their mammalian counterparts, throughout the early embryonic stages (our unpublished observations). However, they play distinct roles in regulating zebrafish gastrulation in that only profilin I is involved and its effects are synergistic with zDia2. This implies that zDia2 works synergistically with Profilin I in mediating cellular movements during gastrulation, possibly via the same signaling pathway. The formins and profilins have been demonstrated previously to be involved in the same signaling pathway. *Caenorhabditis elegans* formin homology protein CYK-1 binds to profilin PFN-1 and regulates the assembly of cortical actin during cytokinesis [26]. It also been shown that *Dictyostelium* Diaphanous, dDia2, interacts preferentially with profilin II and regulates the formation and maintenance of the filapodium by controlling actin turnover at the barbed ends of actin filaments [73]. Furthermore, there is an interesting analogy between Diaphanous and another Profilin binding partner, the Wiskott-Aldrich syndrome protein (WASP). The WASP poses autoinhibition conformation until binding to the active Cdc42 which exposes the binding site to the actin-related protein (Arp)2/3 complex [74], [75]. Actin nucleation is then induced by the WASP-Arp2/3 complex. Profilin seems to synergize with Cdc42 in activating WASP [76]. Here, we showed that zPfnI and zDia2 synergistically regulate actin dynamics in gastrulation cell movements, and the elucidation of activating mechanisms of zPfnI and zDia2 by Rho or Cdc42 awaits further study.

**Figure 11:**
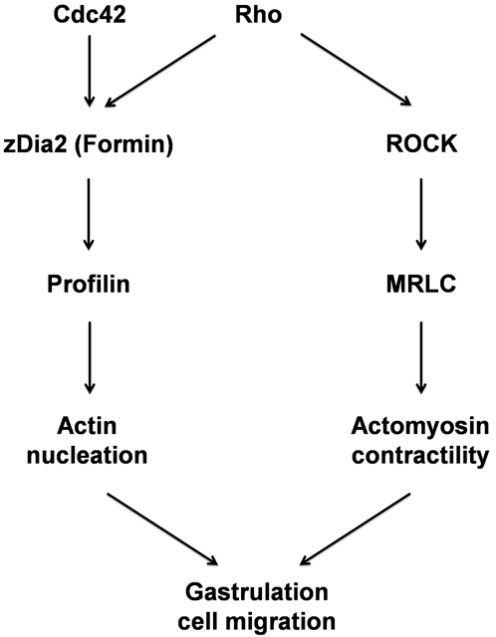
Rho mediates actin remodeling during gastrulation in zebrafish. Rho acts through its downstream effector, Rho-associated kinase (ROCK), which presumably phosphorylates regulatory myosin light chains (MRLCs) and enforces actomyosin contractility. In conjunction, zDia2 or other formins may be activated by Rho and/or Cdc42 to accelerate actin-nucleation through the cooperation with profilin I at the front edge of migrating cells for the control of cellular migration during gastrulation in the zebrafish.

In summary, with the present results and previous findings we have delineated the Rho signaling pathways controlling actin nucleation in gastrulation cell migration and these are illustrated in Fig. 11. During zebrafish development, active Rho leads to the activation of at least two pathways, including ROCK and zDia2-dependent signaling. ROCK phosphorylates its downstream substrate, the regulatory subunit of the myosin light chain, to mediate actomyosin contractility [40], [77], [78]. On the other hand, Rho interacts with zDia2 and zPfnI to nucleate actin at the leading edge of the blastoderm. These two pathways then converge to mediate cellular movements during epiboly and convergent extension in zebrafish gastrulation.

## Materials and Methods

### Maintenance of zebrafish

Breeding fish were maintained at 28.5°C on a 14-h light/10-h dark cycle. Embryos were collected by natural spawning, raised in 0.3× Danieau's buffer (by diluting 1× Danieau's buffer: 58 mM NaCl, 0.7 mM KCl, 0.4 mM MgSO_4_, 0.6 mM Ca(NO_3_)_2_, and 5.0 mM HEPES (pH 7.6), with double distilled water) until observation or fixation. Embryos were staged according to [79], and stages are given as hours post-fertilization (hpf). All animal and embryo handling procedures were approved by the Use of Laboratory Animals Committee at National Taiwan University, Taipei, Taiwan.

### Cloning and analysis of zdia2

A reverse-transcription polymerase chain reaction (RT-PCR) was performed on total RNA extracted from embryos at the designated times using the Trizol reagent (Invitrogen, Carlsbad, CA) according to the manufacturer's instructions. RNA was subsequently treated with DNase I (Invitrogen), and first-strand cDNA was synthesized. cDNA was generated from total RNA of zebrafish larvae at 24 hpf using the SMART RACE kit (Clontech, Mountain View, CA) according to the manufacturer's instructions. PCR primers were designed against a predicted fragment of the *diaphanous* gene (ENSDART00000020945) from Ensemble (http://www.ensembl.org). A 5′ rapid amplification of cDNA ends (RACE) experiment was carried out using the SMART kit-provided 5′ primer and the 3′ primer with the following sequence: 5′-GAAGGWMASRGAMAGCYYGS-3′.

DNA and protein sequences were aligned by tools available at the Biologist's Workbench (http://workbench.sdsc.edu) as described below. Clustal W alignments were prepared to access the identity of the cloned sequence against previously identified homologues in the human and mouse. Domain analysis was performed using HMMPFAM. Phylogenetic analyses were conducted using MEGA version 3.1 [80].

### RT-PCR analysis

RNA and cDNA were prepared as previously described. A 578-bp *zdia2* fragment was amplified with the following primers: 5′-GTMMGMTCTCKYCWCAGGAGT-3′ (forward) and 5′-GAAGGWMASRGAMAGCYYGS-3′ (reverse). Amplification of a 717-bp α-actin fragment served as the RT-PCR control using the following primers: 5′-TTGGTATGGGACAGAAAGACAGCTAC-3′ (forward) and 5′-AAGGGCCACATAGCAGAGCTTC-3′ (reverse).

### Whole-mount in situ hybridization (WISH)

Embryos were grown to desired stages, fixed in 4% paraformaldehyde in phosphate-buffered saline (PBS) overnight, and dechorionated manually by fine forceps. Embryos were then stored in 100% methanol at −20°C until use. Antisense digoxigenin (DIG)-labeled RNA riboprobes were synthesized according to the manufacturer's instructions (Roche Applied Science, Penzberg, Germany). Hybridization and detection with an alkaline phosphatase-coupled anti-DIG antibody (Roche Applied Science) were performed according to Thisse et al. (1993) [81].

### Yeast two-hybrid assay

We identified 3 zebrafish Rho-GTPase genes, including *rhoA* (**BC075938**), *rac1* (**BC044538**) and *cdc42* (**BC057415**), in zebrafish database, cloned and mutated designated amino acids for creations of constitutively-active mutants using QuickChange Site-Directed Mutagenesis Kit (Stratagene, La Jolla, CA) according to the manufacturer's instructions. To determine the interactions of these small GTPases with zDia, we adopted the PROQUEST™ yeast two-hybrid system (Invitrogen), and all procedures were performed according to the manufacturer's instructions. In brief, constitutively active zebrafish Rho-GTPase genes, including G14VRhoA, G12VRac1, and Q61LCdc42 genes, were cloned into the pDBLeu vector, while the 5′ half of *zdia2* of 1487 bp from the start codon was cloned into the pPC86 vector. Active Rho-GTPases in the pDBLeu vector were co-transformed into host yeast cells (strain MaV203) with the 5′half of *zdia2* in the pPC86 vector, respectively. Transformed cells containing both vector constructs were selected with nutrition-deficient medium plates lacking the essential amino acids, leucine and tyrosine. Finally, co-transformed cells were re-streaked onto plated medium lacking histidine with various concentrations of 3-amino-1,2,4-triazo (3AT), and left to grow at 30°C for 2 days. The interaction of the activation domain with the DNA-binding domain induces *HIS3* gene expression which was examined by the growth of yeast cells in medium plates without histidine.

### Morpholino oligonucleotide microinjections

Antisense MOs were purchased or custom made by Gene Tools, LLC (Philomath, OR). A standard control MO (stdMO, 5′-CCTCTTACCTCAGTTACAATTTATA-3′) with no sequence homology to any known zebrafish sequences was used. To knock down the *zdia2* gene activity, we used two different MOs targeting the translation initiation site and the splicing donor of the fourth intron to abolish translation and delete the core Rho-binding domain, respectively. The translation-blocking MO was designated tMO of the following sequence with the target site in parentheses: 5′-AGCTGCCTGCTGATCCATCTTTGGA-3′ (−7 to +18). The splice-blocking MO was designated sMO of the following sequence with the target site in parentheses: 5′-TGTTGTGGTGCACTTACAGATTTGG-3′ (the last 15 bases in exon 4 and first 10 bases in intron 4). To knock down the *profilin* activities, translation-blocking MOs of the following sequence with the target site in parentheses: 5′- CATGACTGCTGATGTCGATCTGAGT-3′ (−22 to +3 of **BC090268**) and 5′-TTCCACGTAGCTTGCCCACGACATG-3′ (−1 to +24 of **BC067152**), were used for *profilin I and profilin II*, respectively. The MOs were dissolved in sterile double-distilled water to 1 mM and stored at −20°C. MOs were diluted to the desired working concentrations in 1× Danieau's buffer with 0.5% phenol red and stored at 4°C before use. Thin-wall (1 mm (o.d.)×0.75 mm (i.d.)) glass capillaries with filaments (A-M Systems, Carlsborg, WA) were pulled using a horizontal puller (P-97, Sutter Instrument, Navato, CA). Embryos at one-cell stage were immobilized at an injection trough on a 100-mm 2% agar plate. MOs were prepared as described at designated concentrations and back-loaded into a pulled capillary. A loaded capillary was forced into the chorion and the yolk cells to reach the junction between the yolk cells and blastodisc where the solution was ejected by using a pressure injector (IM-300, Narishige, Japan). We estimated the injection volume by the clearance of cytoplasm (0.5% of 1-cell yolk volume). After injection, embryos were recovered from the injection troughs and cultured in 0.3× Danieau's buffer at 28.5°C until being examined.

### Measurement and counting of embryos

The numbers of epiboly-defective embryos receiving designated treatments were counted under a stereo microscope and then fixed at the tail bud stage (10 hpf). After WISH staining, embryos with 50% broader notochords in width (according to the *no tail* riboprobe signal) and a prechordal plate-tail bud (*goosecoid*/*no tail* riboprobe signal) angle exceeding 165° were considered to be a convergence and extension defective embryo. All experiments were repeated at least three times.

### Time-lapse epiboly cell migration recording

Embryos receiving designated treatments were dechorionated with 0.67 mg/mL protease (Sigma, St. Louis, MO) and immobilized in 0.8% low-melting agarose (Amresco, Solon, OH) at the sphere stage. The immobilized embryos were recorded from the shield stage. Embryos were observed and the frontier of migrating blastomeres (marginal deep cells) was examined using a 40× water immersion objective under a Leica DM2500 DIC microscope (Leica Microsystems, Wetzlar, Germany). Twenty-min-long movies at 10-sec intervals were then recorded using a CoolSNAP *fx* CCD camera (Roper Scientific, Tucson, AZ). Movies were collected and analyzed using the Simple PCI Imagine System software (Compix, Sewickley, PA). To clearly visualize membrane boundaries, embryos were injected with GFP-GAP43 mRNA, processed as previously described, and examined using a 63× oil immersion objective under a Leica TCS SP5 confocal microscope system (Leica Microsystems, Wetzlar, Germany) at the Technology Commons, College of Life Science, National Taiwan University (TechComm, NTU). In cell tracking experiments after transplantation, transplanted cells were traced by a 1 hr time-lapse recording with 10-sec interval. Movies were then analyzed for migration path, velocity and linearity (the shortest distance between the start and end points of the movement divided by the total distance moved) also by SimplePCI software.

### Filamentous actin staining on whole-mount embryos

To label filamentous actin (F-actin), zebrafish embryos at designated stage were fixed in 4% paraformaldehyde in PBS overnight at 4°C, washed in PBS and manually dechorionated. Embryos were then incubated with rhodamine phalloidin (R-415, 6.6 nM/ml, Molecular Probes, Inc., Eugene, OR) at room temperature for 4–6 hours and then washed intensively with 1% Triton X-100 (PBT). Labeled embryos were then examined using 63× water immersion objective under a Leica TCS SP2 confocal microscope system (Leica Microsystems) at the TechComm, NTU.

### Cell transplantation

For transplantations, donor embryos were injected with a mixture of 0.5% Q-Rhodamine and 8 ng designated MOs. Blastoderm cells of donor embryos were exposed to UV for 30 second to un-cage the Q-Rhodamine and then transplanted into wild type host embryos at sphere stage as described previously [82]. Transplanted cells were detected and further examined by epifluorescent and confocal microscope.

### Statistical analysis

All experimental values are presented as mean±standard deviation and were analyzed by paired-sample Student's *t*-test in Microsoft Excel.

## Supporting Information

Figure S1 Sequence alignment of the spicing variants induced by the zdia2 sMO. Three DNA fragments as shown in Fig. 4B were gel-purified and sequenced by the primer targeting exon 3 as indicated in Fig. 4A. Resultant sequences were aligned using VectorNTI. The dashed regions in the middle of the sequences are nucleotides omitted by splice blocking. The discrepancy of sequences at the first 20 bases was due to sequencing errors.
(0.28 MB TIF)

Figure S2 Knockdown of *zdia2* by MO interferes with convergent extension cell movements. Embryos injected with 10 ng *zdia2* sMO (A and B) or 10 ng stdMO (C and D) were fixed at 6–8 somite stage and stained with *myoD* and *hatching gland* riboprobes by WISH. Photographs were taken from dorsal view (A and C) and side view (B and D).
(3.22 MB TIF)

Figure S3 Knockdown of *zdia2* interferes with protrusion formation at marginal deep cells during epiboly cell movement. Embryos injected with stdMO (A–C) or *zdia2* sMO (D–F) and GFP-GAP43 mRNA were observed under confocal microscope at the 50% epiboly to shield stage. Movies with 15 frame per sec were recorded and selected snapshots from one of the stdMO-injected and *zdia2* sMO-injected embryos movies with DIC channel (A and D), GFP channel (B and E) and overlap of two channels (C and F) are showed here. Blebbing cell processes are indicated by arrowheads.
(3.18 MB TIF)

Figure S4 Knockdown of *zdia2* inhibit actin condensation at the YSL. Embryos injected with 8 ng stdMO (A and C) and *zdia2* sMO (B) were fixed at the germ-ring stage and photographed under confocal microscope after rhodamine phalloidin staining. Ring-like actin condensation (arrows in C) were at the YSL of the stdMO-injected embryo (A and C), but not in the epiboly-defected *zdia2* morphant (B).
(1.04 MB TIF)

Figure S5 Synergistic effect of *zdia2 sMO* and *profilin I* tMO. Embryos were injected with 8 ng *zdia2* sMO (A and B) or co-injected with 4 ng *zdia2* sMO and 4 ng *profilin I* tMO (C and D). Embryos were incubated until tail bud stage (10 hpf), fixed and stained with *ntl* and *gsc* riboprobe. Photographs were taken for side view (A and C) and dorsal view (B and D) after WISH.
(0.73 MB TIF)

Movie S1 Migration of marginal deep cells in a gastrulating embryo injected with stdMO. The marginal deep cells of blastoderm are migrating downwardly toward the vegetal pole.
(4.16 MB MOV)

Movie S2 Migration of marginal deep cells in a gastrulating embryo injected with *zdia2* sMO. The marginal deep cells of blastoderm are migrating downwardly toward the vegetal pole.
(4.06 MB MOV)

Movie S3 The marginal deep cells of blastoderm are migrating upwardly toward the vegetal pole.
(3.68 MB MOV)

Movie S4 The marginal deep cells of blastoderm are migrating downwardly toward the vegetal pole.
(3.43 MB MOV)
