# Supplementary figures and images for: Diaphanous-Related Formin 2 and Profilin I Are Required for Gastrulation Cell Movements
Source: PLoS One. 2008 Oct 21;3(10):e3439. doi: 10.1371/journal.pone.0003439 (PMC2565064; doi:10.1371/journal.pone.0003439)

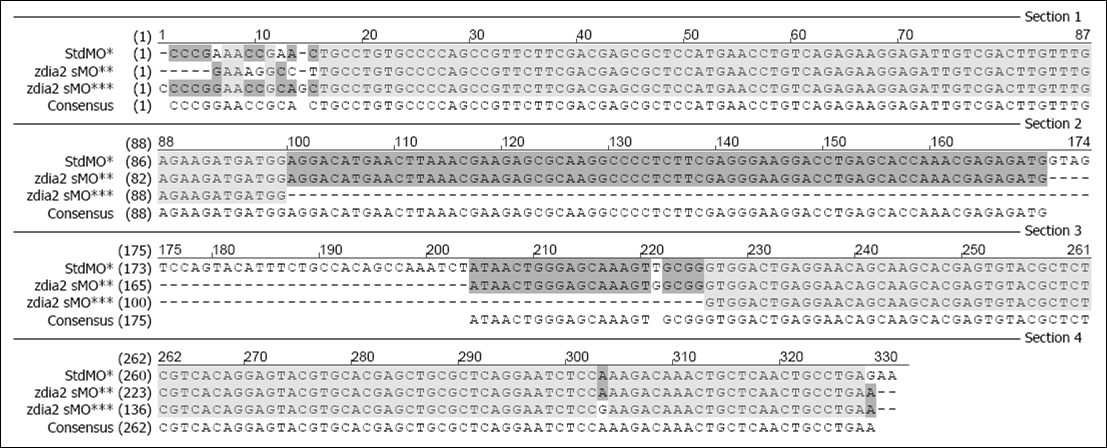

Supplement: Figure S1 — Sequence alignment of the spicing variants induced by the zdia2 sMO. Three DNA fragments as shown in Fig. 4B were gel-purified and sequenced by the primer targeting exon 3 as indicated in Fig. 4A. Resultant sequences were aligned using VectorNTI. The dashed regions in the middle of the sequences are nucleotides omitted by splice blocking. The discrepancy of sequences at the first 20 bases was due to sequencing errors. (0.28 MB TIF) [file pone.0003439.s001.tif]

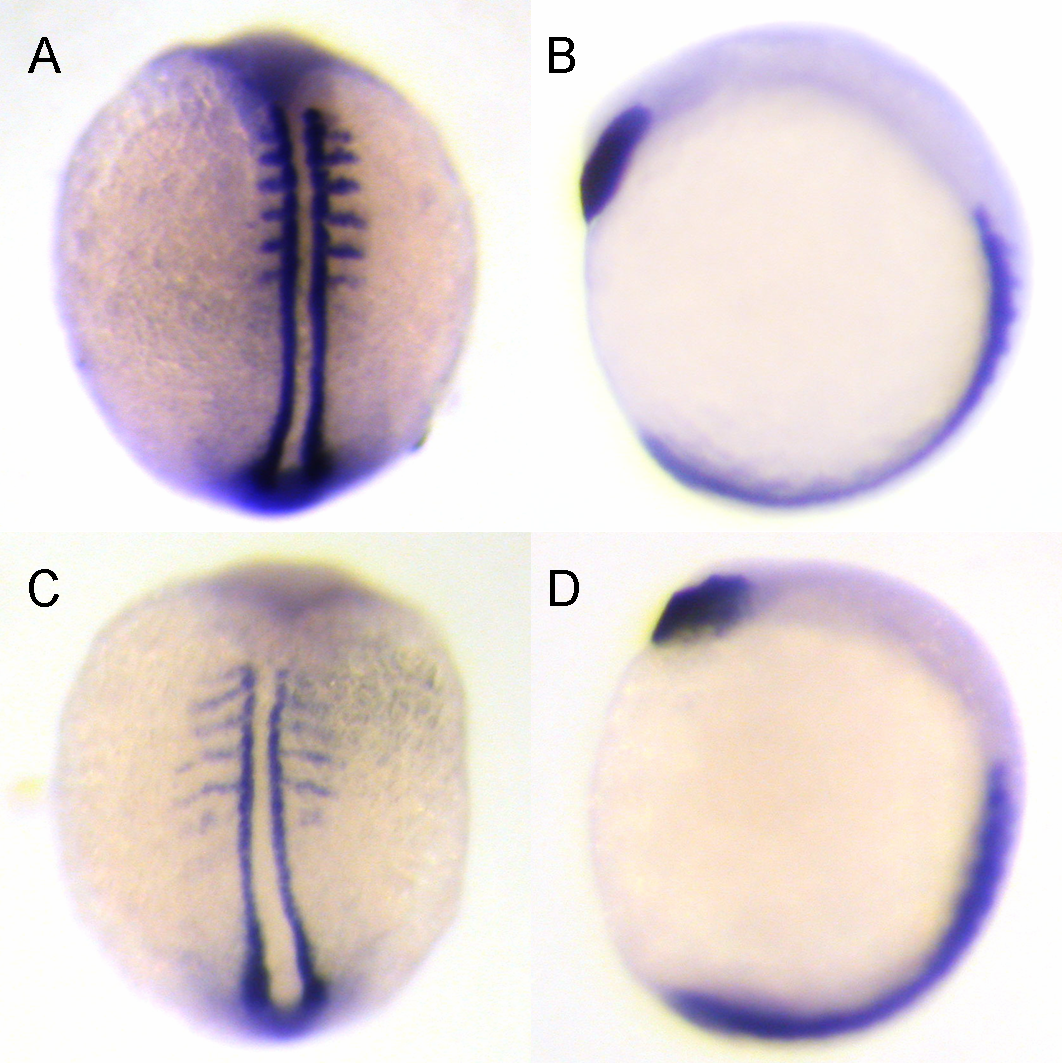

Supplement: Figure S2 — Knockdown of zdia2 by MO interferes with convergent extension cell movements. Embryos injected with 10 ng zdia2 sMO (A and B) or 10 ng stdMO (C and D) were fixed at 6–8 somite stage and stained with myoD and hatching gland riboprobes by WISH. Photographs were taken from dorsal view (A and C) and side view (B and D). (3.22 MB TIF) [file pone.0003439.s002.tif]

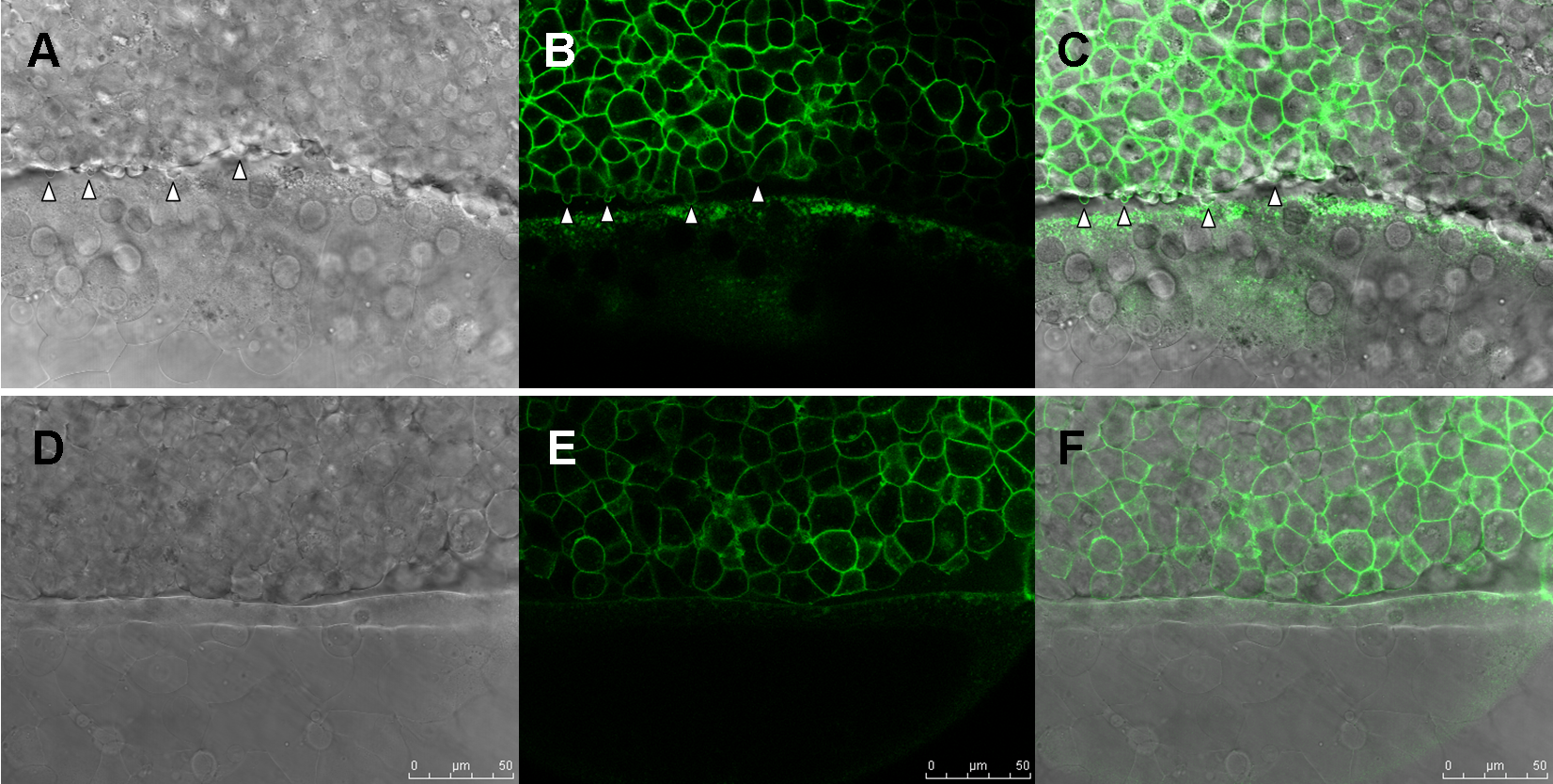

Supplement: Figure S3 — Knockdown of zdia2 interferes with protrusion formation at marginal deep cells during epiboly cell movement. Embryos injected with stdMO (A–C) or zdia2 sMO (D–F) and GFP-GAP43 mRNA were observed under confocal microscope at the 50% epiboly to shield stage. Movies with 15 frame per sec were recorded and selected snapshots from one of the stdMO-injected and zdia2 sMO-injected embryos movies with DIC channel (A and D), GFP channel (B and E) and overlap of two channels (C and F) are showed here. Blebbing cell processes are indicated by arrowheads. (3.18 MB TIF) [file pone.0003439.s003.tif]

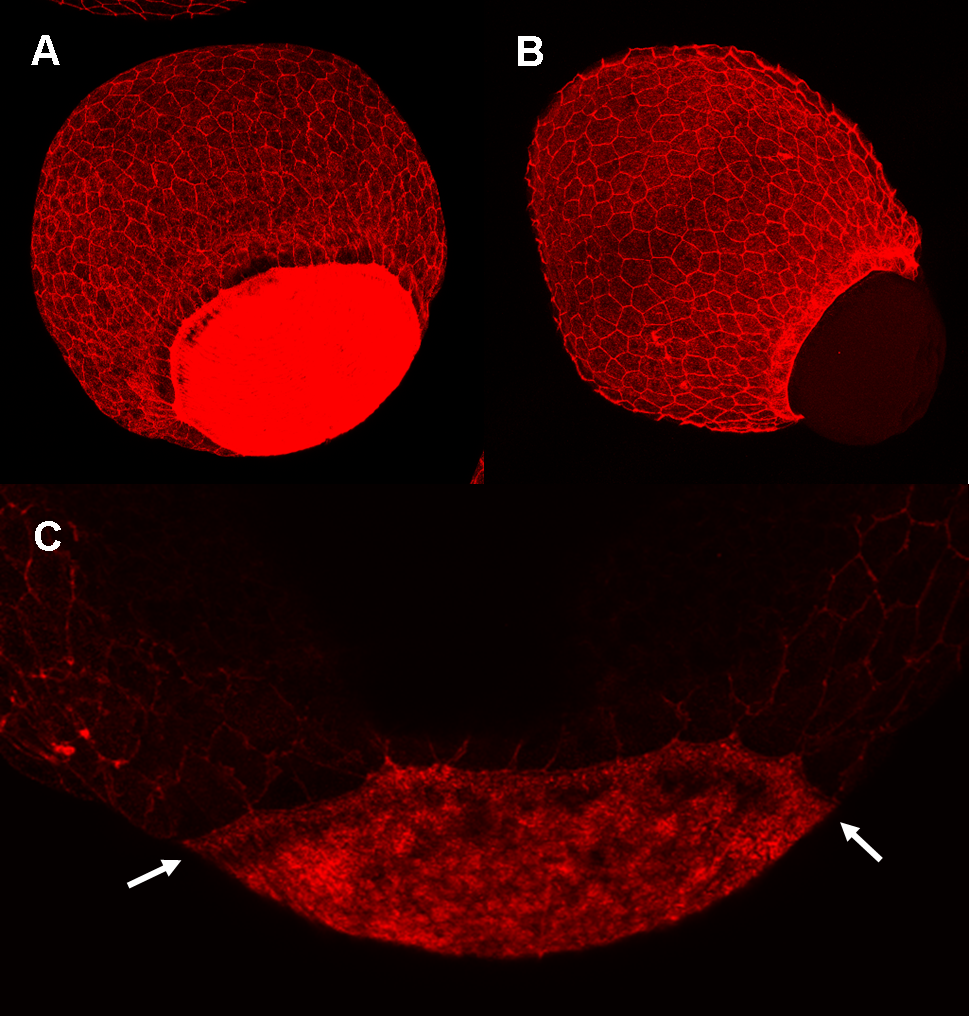

Supplement: Figure S4 — Knockdown of zdia2 inhibit actin condensation at the YSL. Embryos injected with 8 ng stdMO (A and C) and zdia2 sMO (B) were fixed at the germ-ring stage and photographed under confocal microscope after rhodamine phalloidin staining. Ring-like actin condensation (arrows in C) were at the YSL of the stdMO-injected embryo (A and C), but not in the epiboly-defected zdia2 morphant (B). (1.04 MB TIF) [file pone.0003439.s004.tif]

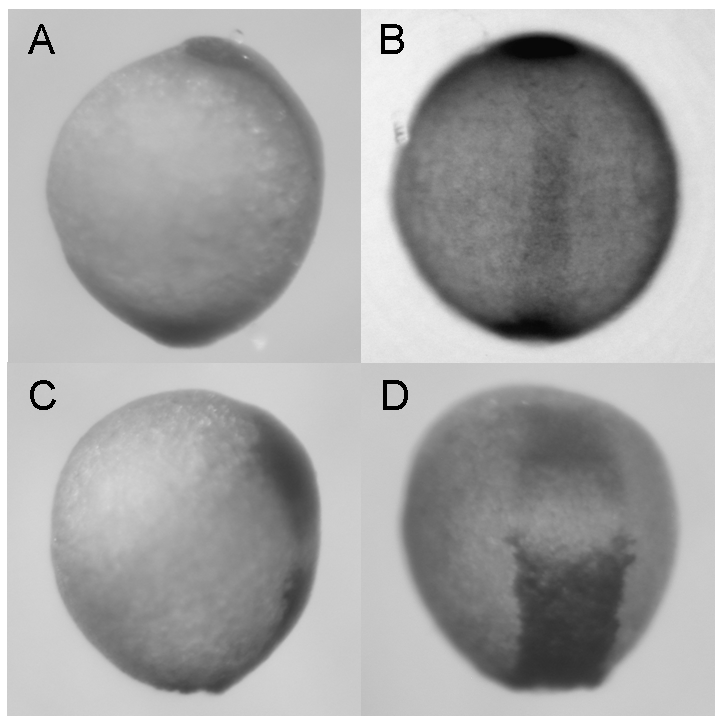

Supplement: Figure S5 — Synergistic effect of zdia2 sMO and profilin I tMO. Embryos were injected with 8 ng zdia2 sMO (A and B) or co-injected with 4 ng zdia2 sMO and 4 ng profilin I tMO (C and D). Embryos were incubated until tail bud stage (10 hpf), fixed and stained with ntl and gsc riboprobe. Photographs were taken for side view (A and C) and dorsal view (B and D) after WISH. (0.73 MB TIF) [file pone.0003439.s005.tif]
